# Preparation, characterization, and pharmacodynamics of insulin-loaded fumaryl diketopiperazine microparticle dry powder inhalation

**DOI:** 10.1080/10717544.2019.1631408

**Published:** 2019-07-01

**Authors:** Yun Xia, Yipeng Su, Qiyue Wang, Chen Yang, Baoqiang Tang, Yue Zhang, Jiasheng Tu, Yan Shen

**Affiliations:** a Center for Research Development and Evaluation of Pharmaceutical Excipients and Generic Drugs, China Pharmaceutical University, Nanjing, China;; b State Key Laboratory of Nature Medicines, Department of Pharmaceutics, China Pharmaceutical University, Nanjing, China

**Keywords:** Insulin, FDKP, diabetic, inhalation, pharmacodynamic

## Abstract

**Purpose:** With the increase of population aging and the proportion of overweight and obese, a growing number of people are suffering from diabetes. Insulin (INS) as the most widely used hypoglycemic agent was always chosen as the most effective treatment method of diabetes. In this study, fumaryl diketopiperazine (FDKP) was used as a carrier for the pulmonary delivery of insulin.

**Patients and methods:** The INS-loaded FDKP microspheres (INS@FDKP-MPs) were prepared by spray drying and physicochemical properties (drug loading, particle size, flowability, moisture content, morphology, and crystalline state) were further investigated. Pharmacodynamics was investigated on diabetic model rats administrated by intratracheal insufflation.

**Results:** The INS-loaded FDKP microspheres show satisfied flowability and *in vitro* deposition with FPF 50.2% and MMAD 3.45** **± 0.13 μm, and the blood glucose level was significantly decreased. Moreover, no inflammatory reaction was observed during the safety study.

**Conclusion:** To sum up, the aim was to develop a non-injection system for insulin, INS@FDKP-MPs powder inhalation with high dose, low toxicity, and good lung deposition inhalation could rapidly decrease the blood glucose level without immune stimulation, which shows remarkably potential on diabetes treatment by pulmonary delivery route.

## Graphical Abstract

**Figure:**
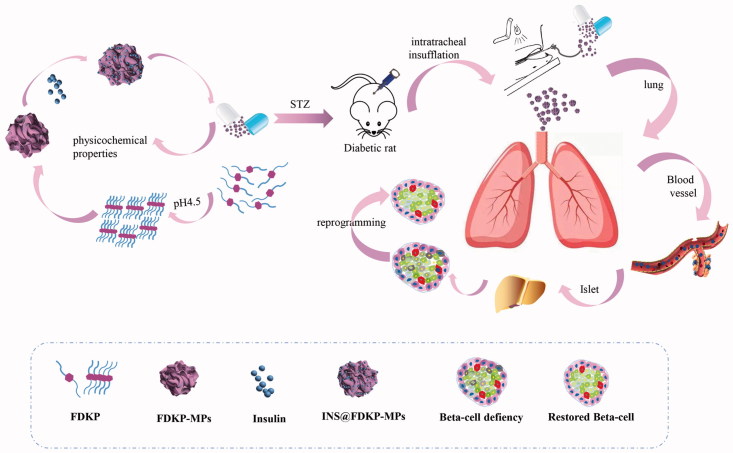

## Introduction

Diabetes mellitus is a common endocrine and metabolic disease caused by the lack of insulin secretion in the body, leading to disorders of protein and fat metabolism caused by hyperglycemia (Olivera et al., 1996). Many studies reported that diabetes is the result of interactions of genetic factors, autoimmune factors, and environmental factors (Qian et al., 2015). At present, the first choice for clinical treatment of diabetes is still insulin (INS) (Nagai et al., 2013), which is secreted by islet beta cells in the pancreas, the only hormone in the body to lower blood sugar. Patients with type 1 or type 2 diabetes mellitus always require insulin therapy to control the blood glucose level every 2 or 3 times per day. So insulin is of vital importance in controlling blood sugar, protecting β cells, preventing and delaying the occurrence of other diseases related to diabetes (Jiang et al., 2018).

The most common therapy method of insulin administration in clinical practice is subcutaneous injection (Hovelmann et al., 2017). While the injection method will not only cause inconvenience to the patient but also cause many side effects such as fat atrophy or fat hyperplasia occurred at the injection site and unsatisfactory blood glucose control (Frid et al., 2016). This highlights the great advantage of non-injection insulin therapy routes for diabetes treatment (Cai & Zhu, 2007; Ishii et al., 2017). The currently known non-injection routes for protein drugs include oral preparations, transdermal patches, pulmonary inhalation administration, and nasal mucosal administration, (Xue et al., 2006; Wong, 2009). However, there still exist many problems in some non-injection routes, because of a large amount of peptide hydrolase and proteolytic enzyme in the gastrointestinal tract, oral administration is easily catalyzed by decomposition, some drugs with relatively high molecular mass have poor permeability and low bioavailability (Zhang et al., 2008; Stegemann et al., 2013). Compared with other non-injection routes, pulmonary administration is more advantageous because of its physiological advantage. It is known that alveoli have the characteristics of large surface area, high permeability, and large circulation perfusion, when the protein drug is inhaled into the deep lung, the drug can be quickly absorbed into the blood circulation, it not only prevents macromolecular drugs from being decomposed by digestive enzymes in the intestine but also avoids the ‘first pass effect’ of the liver (Harsch, 2005), thus pulmonary delivery of drugs for both local and systemic action has gained new attention over the last decades (Andrade et al., 2015). Dry powder inhalers (DPIs) as a form of pulmonary administration were frequently selected as the optimized route for insulin pulmonary delivery (Chan, 2006; Wang & Lei, 2011). It is reported that co-spray drying has been widely used in the DPI production, it can offer an easily controlled particle formulation, create special structure (e.g. porous particles), and appropriate morphology of the API because of the scalability (Healy et al., 2014; Chvatal et al., 2019).

Pharmaceutical excipients were always used in the formulation of dry powder inhalation to improve flowability, moisture resistance, and deposition (Pilcer & Amighi, 2010; Healy et al., 2014). Lactose monohydrate is the most commonly used excipient carrier material in DPI formulations (Pilcer et al., 2012). Unfortunately, the degradation of lactose may have an effect on the stability of peptide drugs, it may cause many uncertainties, in addition, lactose as a carrier is not suitable for patients with diabetes and those who are allergic to lactose (Zeng et al., 2000; Hickey, 2003). Others, such as phospholipids, bulking agents, and biodegradable polymers (e.g. PLGA), could eventually lead to adverse immune responses in the lungs resulting in cough, edema, and neutrophil infiltration (Wang et al., 2018).

Towards this end, [Bis-3,6(4-fumarylaminobutyl)-2,5-diketopiperazine] (FDKP) is an FDA approved inert excipient that has been used as the primary component in Afrezza^®^ to assist in the delivery of recombinant human insulin *via* inhalation (Rendell, 2014; Maker et al., 2017). As a new excipient, FDKP is chemically inert and can be assembled into microspheres by hydrogen bonding under acidic conditions and dissolves under neutral and alkaline conditions (Angelo et al., 2009). The microspheres could adsorb the insulin through electrostatic interactions to form microparticles in a range between 0.5 and 10** **μm, more specifically 3–5** **μm (geometric particle size and MMAD) for insulin particles (Liu et al., 2008). Absorbed FDKP is directly excreted from the kidneys without metabolism which avoiding the toxicity of degradation products. Although the inhaled insulin powder Afrezza^®^, which has been marketed, can significantly reduce the incidence of hypoglycemia and relieve the pain of patients, the sales volume and the market feedback of Afrezza^®^ is unsatisfactory as well. The reason for the poor sales of Afrezza^®^ may be that people are still skeptical about the safety of insulin DPIs, and the price of the inhaler and its associated inhalation device is higher than that of insulin injection (Fantasia, 2015). Compared with Afrezza^®^, this paper aims to optimize the preparation process of INS@FDKP-MPs by adopting a simpler and more convenient synthesis method to reduce the production cost, and by evaluating the properties of INS@FDKP-MPs, it is intended to construct an inhalable insulin preparation that is qualified in particle size, drug loading, hypoglycemic effect, and safety.

In this article, insulin was used as a model drug and that could be administered deep into the lungs (Potocka et al., 2010). Recombinant human insulin was adsorbed on FDKP crystal particles, INS@FDKP-MPs pulmonary inhalation powder was prepared and then its physical and chemical properties were investigated. The experiment of formulation factors on the properties of INS@FDKP-MPs (e.g. polysorbate 80 concentration, centrifugation speed, stirring speed, homogenization time, pressure) and orthogonal design optimization of spray drying process (e.g. pump speed, air input, aspirator inlet temperature) were then conducted to optimize the preparation process of INS@FDKP-MPs. The toxicity of the INS@FDKP-MPs to the lungs was studied by investigating the pharmacodynamics of INS@FDKP-MPs *in vivo* to achieve rapid release into the blood circulation to play a hypoglycemic effect with high dose, low toxicity, and good lung deposition inhalation powder aerosol agent finally.

## Material and methods

### Materials and animals

Recombinant human insulin (titer: 27.5** **U** **mg^−1^, lot number: 0512A04) was purchased from Wanbang Biopharmaceuticals Co., Ltd. (Xuzhou, China) and insulin reference (lot number: 140633-201406) was obtained from National Institutes for Food and Drug Control (Beijing, China). Fumaryl diketopiperazine (FDKP) was synthesis by the laboratory. Polysorbate 80 (injection grade) was supplied from Nanjing Well Pharmaceutical Co., Led (Nanjing, China). All other reagents were purchased from the Sinopharm Chemical Reagent Co., Ltd and were of analytical grade.

Healthy male SD rats weighing 200** **±** **20** **g and normal mice weighing 20** **±** **2** **g were both purchased from the Experimental Animal Center of Nanjing Qinglongshan. All animal experiments were conducted in accordance with the Guide for Laboratory Animal Facilities and Care and were approved by the Animal Ethics Committee of China Pharmaceutical University.

### Synthesis of FDKP

FDKP was synthesized using a three-step reaction as previously described and was summarized in the Supporting Information section (Wang et al., 2018) (Figure S1). The chemical structure of the resulted FDKP was identified by ^1^H NMR, IR spectrum (TENSOR 27, Bruker, Germany) and mass spectrometry (Bruker Amazon ETD, Germany).

**Figure 1.**
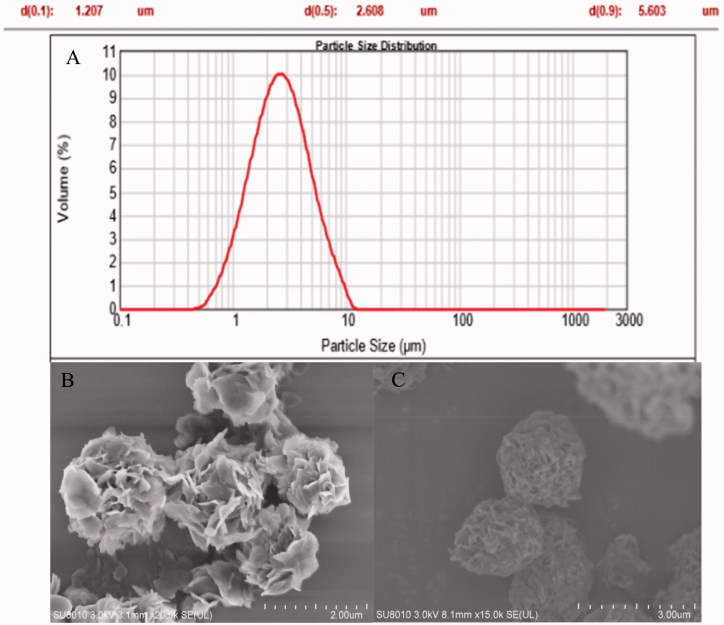
(A) Profile of particle distribution of FDKP-INS microsphere dry powder inhalation; spray-dried powder morphology visualized by scanning electron microscopy; (B) blank microsphere dry powder inhalation; (C) INS@FDKP-MPs microsphere dry powder inhalation.

### Preparation of ISN-loaded FDKP microparticle powder (ISN@FDKP-MPs)

INS@FDKP-MPs were prepared by spray drying (Mini Spray Dryer B-290, Buchi, Switzerland). Two hundred and fifty milligrams of FDKP was dissolved in 10** **mL of ammonia aqueous solution (1%, v/v) containing 0.3% (w/w) polysorbate 80. Ten microlitres of acetic acid solution (10%, v/v) also containing 0.3% (w/w) polysorbate 80 was added dropwise into FDKP solution with magnetic stirrer and adjust pH to 4.5 with FDKP precipitated progressively (Wang et al., 2018; Shi et al., 2012). FDKP suspension was further homogenized for 9** **min at pressure 140** **bar to prepare FDKP microparticle. Forty milligrams of insulin was precisely weighted, dissolved in 20** **mL of 2% (w/w) acetic acid solution, and added to the FDKP microparticle suspension with flow rate 2.0** **mL** **min^−1^ and further stirring for 2** **h to obtain INS@FDKP-MPs, it was stored in a refrigerator at 4** **°C.

### Effects of formulation factors on the properties of INS@FDKP-MPs

Five groups of three batches of INS@FDKP-MPs were prepared, and the effects of polysorbate 80 concentration (0.1, 0.2, and 0.3%), centrifugation speed (3000, 4000, and 5000** **rpm** **min^−1^), stirring speed (100, 300, and 500** **rpm** **min^−1^), high pressure homogenization time (5, 7 and 9** **min), and high pressure homogenization pressure (120, 130, and 140** **bar) on the morphology, dumping rate of the microspheres, and particle size of drug-loaded microspheres were investigated.

### Orthogonal design optimization of spray drying process for the preparation of INS@FDKP-MPs

The pump speed (A), air input (B), aspirator (C), and inlet temperature (D) were selected as the factors to be investigated during the spray drying process. Three levels were selected for each factor, L_9_ (3^4^) orthogonal table is shown in Table S1. Nine batches of INS@FDKP-MPs were prepared according to the orthogonal test table, the yield, aerodynamic diameter, moisture, and angle of repose were taken as the main indicators and *Z*-comprehensive scoring method (Becker & Frick, 2008) was used to evaluate and select the optimal spray drying preparation process.

**Table 1.** The factor of polysorbate 80, centrifugation speed, stirring speed, homogenization time, and homogenization pressure (*n* = 3).

Variable		Morphology	Dumping rate	Size (μm)
Polysorbate 80 (%)	0.1	Irregular	>90%	9.76 ± 1.16
	0.2	Moderate	Good	4.23 ± 0.22
	0.3	regular	Good	6.71 ± 0.43
Centrifugation speed (rpm min^−1^)	3000	regular	Good	5.34 ± 1.35
	4000	regular	Good	4.89 ± 0.94
	5000	Moderate	Poor	5.13 ± 0.66
Stirring speed (rpm min^−1^)	100	regular	Poor	6.34 ± 1.71
	300	regular	Good	2.33 ± 0.54
	500	Irregular	>80%	3.95 ± 0.50
Homogenization time (min)	5	Moderate	Poor	5.85 ± 1.46
	7	regular	Good	4.91 ± 0.97
	9	regular	Good	3.76 ± 1.69
Homogenization pressure (bar)	120	Moderate	>70%	8.96 ± 1.57
	130	regular	Moderate	5.31 ± 2.03
	140	regular	Good	3.86 ± 1.32

### Insulin content assay

INS@FDKP-MPs were collected by centrifuging suspension at 4000** **rpm** **min^−1^ for 10** **min. The microspheres were dissolved in a certain volume of PBS buffer (pH 7.4), and diluted 10 times to measure the concentration of insulin by HPLC.

The HPLC system (DIONEX Ultimate 3000, Thermo Fisher, Waltham, US) were equipped with a Quaternary Pump, an Online Degasser, an Autosampler and a UV Detector. Data acquisition was performed by the Chromeleon software after the experiments. The analysis was carried out at 214** **nm with a Sepax Bio-C_18_ reversed-phase column of 250** **mm** **×** **4.6** **mm i.d., 5** **μm dimensions. The column temperature was kept at 40** **°C and the injection volume was 20** **μL. The mobile phase was a 74:26 mixture (v/v) of sulfate buffer (0.05** **mol** **L^−1^ Na_2_SO_4_, 0.025** **mol** **L^−1^ NaH_2_PO_4_, and adjust the pH to 2.3 with H_3_PO_4_) and acetonitrile, the flow rate was 1.0** **mL** **min^−1^.

Record the total dose (*W*_T_), the free drug amount (*W*_F_), and the total mass of the microspheres (*W*_M_) and calculated the drug loading (DL %) of the microspheres according to following equation:

(1) $$\mathrm{DL}\%=\;(W_{T}-W_{F})/W_{M})\;\times \;100\%$$

### Particle size, density, and moisture content

The particle size of INS@FDKP-MPs dry powder was measured by powder laser particle size analyzer (BT2001, Jilin better instrument, China).

The density was measured as the following process. A certain amount of INS@FDKP-MPs dry powder was filled into a 5** **mL cylinder with volume recorded as *V*_0_ and then tapped mechanically until no volume change was observed. Record the volume (*V*_1_) and calculated bulk density (BD) and tapped density (TD) according to the following equation:

(2) $$\mathrm{BD}=\frac{\text{Weight of powders}}{V_{0}}$$

(3) $$\mathrm{TD}=\frac{\text{Weight of powders}}{V_{1}}$$

The angle of repose (*α*) and Carr's index were both used to characterize the flowability of dry powders. The angle of repose (*α*) was determined by passing a fixed amount of powders through a funnel that was set at a predetermined height and was calculated according to Equation (4):

(4) $$\text{tan}\left(\alpha \right)=\frac{\mathrm{Height}}{\text{radius of cone}}$$

Carr's index was calculated according to Equation (5):

(5) $$\mathrm{Carr}’\text{s index}=\frac{\mathrm{TD}-\mathrm{BD}}{\mathrm{TD}}\times 100\%$$

The theoretical aerodynamic diameter (*D*_aer_) of INS@FDKP-MPs were calculated according to Equation (6):

(6) $$D_{\mathrm{aer}}=\sqrt{\frac{\mathrm{TD}}{\rho }\times D}$$

In which *ρ*** **=** **1** **g** **cm^3^, *D* was the geometric diameter and TD was the tapped density.

The moisture content of INS@FDKP-MPs was measured by a micro-moisture analyzer (KLS-411, Shanghai Precision Scientific Instruments Co., Ltd., China) using Karl Fischer's moisture measurement method.

### Scanning electron microscopy (SEM)

The shape and surface morphology of INS@FDKP-MPs dry powder was investigated by SEM (Hitachi SU8010, Hitachi Limited, Japan). The powders were stuck on a silicon stub by double sided tape, spray with gold for 200** **s by Hitachi ion sputter coater (E1010, Hitachi Limited, Japan) and then observed with operating voltage at 15** **kV.

### X-ray powder diffraction (XRPD)

XRPD patterns of INS, FDKP, physical mixture, FDKP-MPs, and INS@FDKP-MPs were investigated with an X-ray powder diffractometer (D8 Advance, Bruker^®^, Germany). The powders were filled in the horizontal square recess of a sample holder and excess powders were removed. The surface of the powder samples should be smooth and level with the sides of the holder by flattening gently with the razor blade. The scan was performed at 2.00°** **min^−1^ over a 2*θ* range of 3.0°–40.0°.

### Differential scanning calorimetry (DSC)

Thermal phase transitions of the particles were analyzed by DSC (DSC 204 HP NETZSCH^®^, Germany). Certain amounts of INS, FDKP, physical mixture, FDKP-MPs, and INS@FDKP-MPs were filled into an aluminum sample pan which was then hermetically sealed. The powder samples were heated from 40 to 500** **°C with increasing rate of 10** **°C** **min^−1^ and ultra-high pure nitrogen as the purging gas.

### Evaluation of aerosol dispersion performance in vitro by the next generation impactor (NGI)

The atomization performance of INS@FDKP-MPs dry powder was investigated using a self-made atomization device. A hydroxypropyl methylene cellulose (HPMC) capsule (5^#^, Capsugel^®^ Lonza, Basel, Switzerland) contains about 10** **mg of INS@FDKP-MPs dry powders were install on the atomization device with aurilave to simulate atomization energy source. The dispersion behaviors of dry powders were recorded by a digital camera with a time interval of 0.02** **s.

*In vitro* dispersion performance of INS@FDKP-MPs dry powders was measured by the next generation impactor (NGI, Copley Scientific Limited, Nottingham, UK) equipped with a stainless steel induction port and a USP throat. The HPMC capsules contain about 10** **mg INS@FDKP-MPs were install in the DPI (DL-D01, Charamedical, Tai An, China) and INS@FDKP-MPs were discharged into NGI with an air flow rate 60** **L** **min^−1^ for 4** **s. The powders deposited on the stages of NGI were dissolved in the PBS (pH 7.4) and analyzed for INS concentration by HPLC (Utimate 3000, Thermo Scientific) with a RP-C_18_ column (Sepax Bio-C_18_, 5** **μm, 250** **mm** **×** **4.6** **mm, 300** **A). The INS was detected by measure the absorbance at a wavelength of 214** **nm with the mobile phase (0.05** **M Na_2_SO_4_ and 0.025** **M NaH_2_PO_4_: acetonitrile (74:26, v/v, pH 2.3)) flow rate 1** **mL** **min^−1^. The emitted fraction (EF), fine particle fraction (FPF), and respirable fraction (RF) were calculated by Equations (7)–(9):

(7) $$\mathrm{EF}\left(\%\right)=\frac{\mathrm{ED}}{\mathrm{TD}}\times 100\%$$

(8) $$\mathrm{FPF}\left(\%\right)=\frac{\mathrm{FPD}}{\mathrm{ED}}\times 100\%$$

(9) $$\mathrm{RF}\left(\%\right)=\frac{\mathrm{FPD}}{\mathrm{DD}}\times 100\%$$

where total dose (TD) is the amount of powders loaded in the capsules; emitted dose (ED) refers to the dose discharged from the capsules; fine particle dose (FPD) is the dose deposited on stage 2–7; and deposited dose (DD) represents the dose deposited on impactor stage 1–7.

The mass median aerodynamic diameter (MMAD) and the geometric standard deviation (GSD) of INS@FDKP-MPs were calculated bead on NGI data using an online MMAD calculator (2015 Edition).

### Pharmacodynamic study of ISN@FDKP-MPs in a rat model of diabetes

Type I diabetic rat model was established by intraperitoneal injection of streptozotocin (STZ) solution. STZ was dissolved by the citric acid buffer solution (pH 4.5) and diluted to STZ concentration was 1% (w/w). SD rats have fasted for 12** **h, then STZ solution was administrated by intraperitoneal injection with a dose of 65** **mg** **kg^−1^. The blood glucose was measured 72, 96, and 120** **h after injecting to confirm the establishment of the diabetic model.

Thirty diabetic model rats were randomly divided into five groups (*n*** **=** **6). (A) control group; (B) insulin administrated by injection (5** **U** **kg^−1^); (C–E) ISN@FDKP-MPs administrated by intratracheal insufflation with low (5** **U** **kg^−1^), medium (10** **U** **kg^−1^), and high (20** **U** **kg^−1^) dose. For Groups A and B, saline and insulin injection were administrated by subcutaneous injection separately. For Groups C–E, rats were anesthetized by intraperitoneal injection of sodium pentobarbital (3%) with dose 40** **mg** **kg^−1^ and fixed on an oblique plane at an angle of 60°. A cannula tube (18** **G, 3** **cm) was inserted into the tracheal with the help of an endoscope and the FDKP-INS dry powder (5, 10, and 20** **U** **kg^−1^) were insufflated with the help of a syringe to ensure the delivery of all powders into the lung. The blood of rats were collected from the caudal vein at 5, 10, 20, 30, 45, 60, 90, 120, 180, 240, and 360** **min after administration, respectively, and the concentration of blood glucose was measured with Johnson & Johnson Blood Glucose Meter (ONE TOUCH Ultra, Johnson & Johnson, New Brunswick, US).

### Safety of ISN@FDKP-MPs

A total of 12 normal mice were randomly separated in to four groups (*n*** **=** **3). Group 1 was an insulin-free FDKP blank powder to investigate whether FDKP had damage to rat lung tissue (Blank DPIs); Groups 2 and 3 were respectively INS@FDKP-MPs group and common lactose insulin powder group (Common INS) administered at a dose of 20** **U** **kg^−1^ (Courrier et al., 2002); Group 4 was the subcutaneous insulin injection group (5** **U** **kg^−1^) as a negative control group for lung tissue damage (*s.c.*). All the mice were sacrificed at 24** **h after the administration and lungs were immediately harvested and fixed in 4% (v/v) of paraformaldehyde solution at 4** **°C for 48** **h. Then the lungs were transferred to ethanol, xylene, and paraffin, respectively, for the dehydration and paraffin embedding. Micron sections were prepared, stained with hematoxylin and eosin (H&E), and further examined by a light microscope. The images were acquired using a JFMV300CG camera and JFMV controller software (Nan Jing Ji Fei Technology Co., Ltd., Nan Jing, China).

### Statistical analysis

All experiments were conducted in triplicate and repeated at least three different times. Statistical differences between means were determined by SPSS (version 17.0, IBM, Armonk, US) using the one-way ANOVA, where *p**** ***<*** ***.05 was considered statistically significant. All values are reported as mean** **±** **SD.

## Results

### Synthesis and characterization of FDKP

The structure of the product was confirmed by ^1^H NMR, IR, and ESI-MS, as shown in the Supporting Information section (Figure S2).

**Figure 2.**
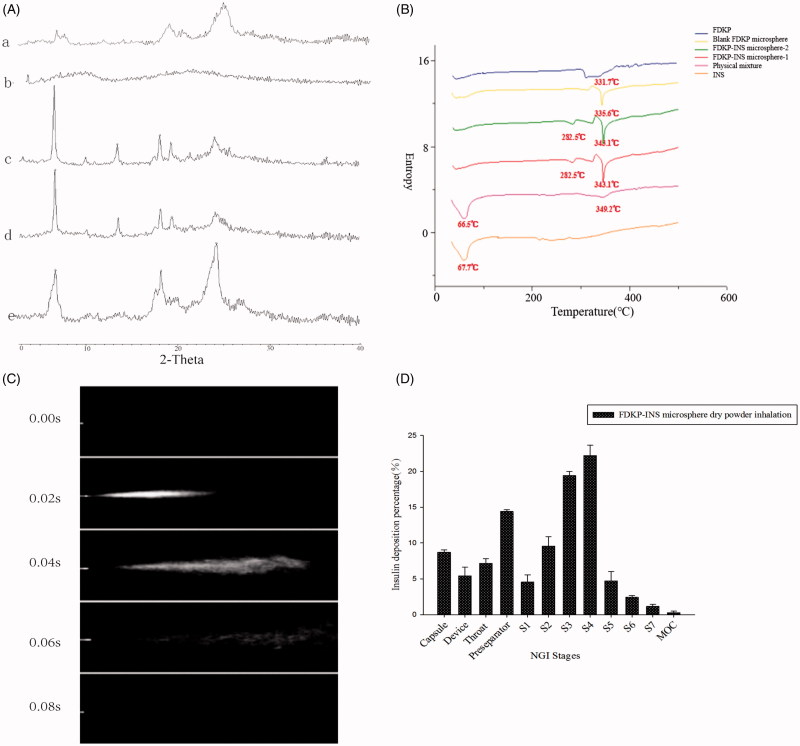
(A) X-ray diffraction patterns of (a) INS@FDKP-MPs dry powder inhalation; (b) insulin raw material; (c) FDKP before spray drying; (d) physical mixture; (e) FDKP after spray drying; (B) DSC diagrams; (C) delivery sequence of FDKP-INS microsphere dry powder inhalation during the actuation of self-made inhalation atomization device; (D) INS@FDKP-MPs dry powder inhalation aerosolization by NGI.

### Optimization of optimal process conditions

According to the analysis of the results (Table 1), when the polysorbate 80 concentration was 0.3%, the centrifugal speed was 4000** **rpm** **min^−1^, the stirring speed was 300** **rpm** **min^−1^, the high-pressure homogenization time was 9** **min, and the high-pressure homogenization pressure is 140** **bar, the drug-loaded microspheres with small particle size, good shape, and good dispersibility can be obtained.

### Results of spray-drying orthogonal experiment for the preparation of INS@FDKP-MPs

The aerodynamic diameter, yield, moisture content, and angle of repose of each batch of DPI are shown in Table S2. The calculation methods of the *Z*-comprehensive scoring method (Table S3) show that the order of influence of the four process factors on the INS@FDKP-MPs inhalation powder is: D** **>** **A** **>** **B** **>** **C. The orthogonal test results show that the optimal spray drying process is A1B2C3D1. INS@FDKP-MPs were obtained with the following conditions: inlet temperature of 140** **°C, pump speed at 100% (35** **m^3^** **h^−1^), air input at 533** **L** **h^–1^, and aspirator (%) at 4% (1.75** **mL** **min^−1^).

**Table 2.** Determination of insulin in FDKP-INS microsphere dry powder inhalation.

No.	Yield (%)	Insulin loading (%)
1	53.2	96.4
2	49.6	97.2
3	55.1	96.7
Average	52.6 ± 2.79	96.8 ± 0.4

### Insulin content determination methodology

Calibration graphs were constructed in the range 10–120 μg** **mL^−1^ for insulin. The regression equations of these curves and their coefficients of determination (*r*^2^) were calculated as follows: *y*** **=** **0.1874*x*** **+** **0.0083 (*r*^2^** **=** **0.9997). The microspheres prepared under the process conditions had a drug loading of 13.23% and an aerodynamic diameter (*D*_aer_) of 1.136** **μm.

### Physicochemical properties of INS@FDKP-MPs

The particle size, size distribution, repose angle, and Carr's index of INS@FDKP-MPs dry powders were characterized. High spray-drying yield was found about 52.60** **±** **2.79% with entrapment rate over 96.8** **±** **0.4% which represent that almost all the insulin were loaded on the FDKP microparticles (Table 2). The particle size was measured by the laser particle size analyzer and the *D*_aer_ was calculated as 1.566** **±** **0.034** **μm, which indicated the satisfied potential of lung deposition performance (Figure 1(A)).

To characterize the flowability of the dry powders, repose angle, and Carr’s index were measured and calculated through bulk density (0.224** **g** **cm^3^) and tap density (0.286** **g** **cm^3^). Repose angle of INS@FDKP-MPs was 33.1° lower than 35° which met the requirements of the industrial production process and the Carr’s index also shows satisfied flowability with value 21.7% smaller than 22%.

The moisture content of the INS@FDKP-MPs dry powder was lower than 1.8% after spray drying and the lower water content was favorable for the insulin stability and for the aerosol performance of dry powder inhalation.

### Scanning electron microscopy (SEM)

The particle size and surface morphology of INS@FDKP-MPs was visualized by SEM. As shown in Figure 1(B–C), the size of the blank microparticles and drug-loaded microparticles was all below 5** **μm, which was consistent with the determination data of particle size analyzer. The blank microparticle shows a porous petal-like structure which could significantly improve the dispersion performance. INS@FDKP-MPs shows soother surface as insulin covered on the blank microparticles through electrostatic adsorption.

### X-ray powder diffraction (XRPD)

XRPD was used to investigate the crystallinities of INS@FDKP-MPs dry powder. Insulin shows no obvious diffraction peak which indicated an amorphous state was existed after the spray drying (Figure 2(A)). FDKP raw materials show sharp diffraction peak at 2*θ***  **=** ** 7°, 18°–19°, and 25° drying by the oven which existed as crystalline state, while the diffraction peak in 2*θ***  **=** **7°, 18°–19° were significantly decreased and only the peak in 2*θ*** **=** **25° still can be recognized after spray drying to prepare blank FDKP microparticles which might indicate FDKP forming different crystal type when drying at the different temperature. The INS@FDKP-MPs show same diffraction peak with FDKP microparticles spray dry powders with characteristic diffraction peak at 2*θ*** **=** **25° and small peaks at 2*θ*** **=** **7° and 18°–19°, which indicate spray drying with insulin will not affect the crystal type of FDKP.

### Differential scanning calorimetry (DSC)

DSC was also used to investigate the crystallization behaviors of INS@FDKP-MPs. As shown in Figure 2(B), insulin shows an endothermic peak around 67** **°C which is caused by the melting behavior. FDKP raw materials show an obtuse endothermic peak around 320–330** **°C. While after spray drying, the endothermic peak transferred to around 334** **°C and indicate that the crystal type might have changed during higher drying temperature which is also consistent with the XPRD data. INS@FDKP-MPs spray dry powder shows an endothermic peak at 343** **°C but the peak of insulin disappeared as a complex was formed with FDKP with a new peak appeared around 282.5** **°C.

### *In vitro* aerosol dispersion performance

Visualized dispersion images were obtained by using a digital camera to shoot a serious of aerosol patterns after instillation by self-made atomization device. The INS@FDKP-MPs could rapidly be atomized and dispersed to form the conical-like homogeneous aerosol which exhibits strong atomization ability (Figure 2(C)).

NGI was used to further investigate the dispersion performance and *in vitro* deposition of INS@FDKP-MPs. As shown in Figure 2(D), residual dry powder in the capsule was less than 10% which indicated that INS@FDKP-MPs shows satisfied emit ability and could be easily aerosolized and passed through the device. What is more, the fine particle fraction (FPF) and the respirable fraction (RF) of the INS@FDKP-MPs were respectively found to be 50.2% and 52.1%. The dry powder inhalation shows high deposition in stage 3 (4.46–2.82** **μm) and 4 (2.82–1.66** **μm) with MMAD 3.45** **±** **0.13** **μm indicating that the powder could be deposited into deep lung with satisfied dispersion performance.

### Pharmacodynamic study

STZ solution was administrated to the normal rats by intraperitoneal injection and the blood glucose level was measured to confirm the successful establishment of diabetic rat model. Starting from the third day after STZ solution injection, the blood glucose level increase up to 20** **mmol** **L^−1^ and maintained more than 3** **days higher than 16.7** **mmol** **L^−1^, which confirmed the diabetic rat model was successfully established.

**Figure 3.**
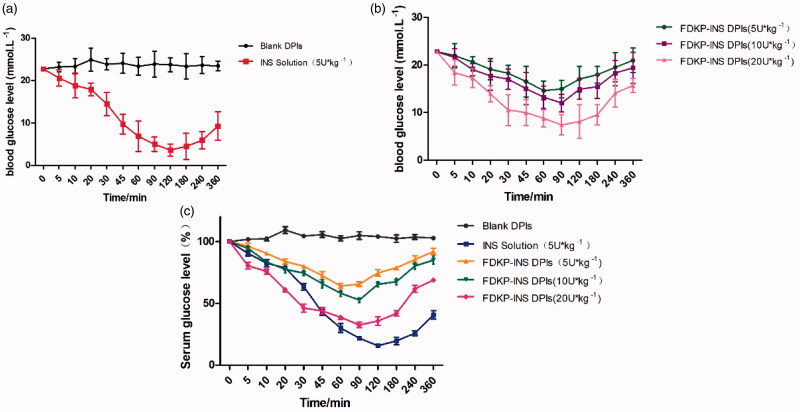
(a) Blood glucose level–time curve of diabetes rats that inhaled blank dry powder inhalation and subcutaneous injected insulin solution (5 U kg^−1^); (b) blood glucose level–time curve of diabetes rats that inhaled FDKP-INS dry powder inhalation for the dose of 5, 10, 20 U kg^−1^; (c) percentage–time curve of blood glucose in rats after administration of different carriers. Each point presents the mean ± SD (*n* = 6).

Diabetic rats were further used to investigate the hypoglycemic effect of INS@FDKP-MPs. The results indicated that the Blank DPIs did not affect the blood glucose level of rats. While the blood glucose level of the subcutaneous injection group greatly decreased to 15.8% and maintained the level below 50% after injecting for 360** **min, which easily lead to the side effect like hypoglycemia (Figure 3(a)). Compared to the subcutaneous route, administration of INS@FDKP-MPs by intratracheal insufflation also could rapidly decrease the blood glucose concentration 5** **min after the administration. An increase of delivery dose could not only promote the hypoglycemic effect with lowest relative blood glucose concentration decreased from 64.2% to 32.6%, but also leading to the delay of *T*_min_ from 60 to 90** **min when dose increases from 5 to 20** **U** **kg^−1^(Figure 3(b)). Administration INS@FDKP-MPs by intratracheal insufflation, the hypoglycemic effect could maintain 120** **min with glucose concentration keep decreasing which was significantly shorter than the subcutaneous injection (180** **min) which reduce the possibility of hypoglycemia (Figure 3(c)).

### Safety

**Figure 4.**
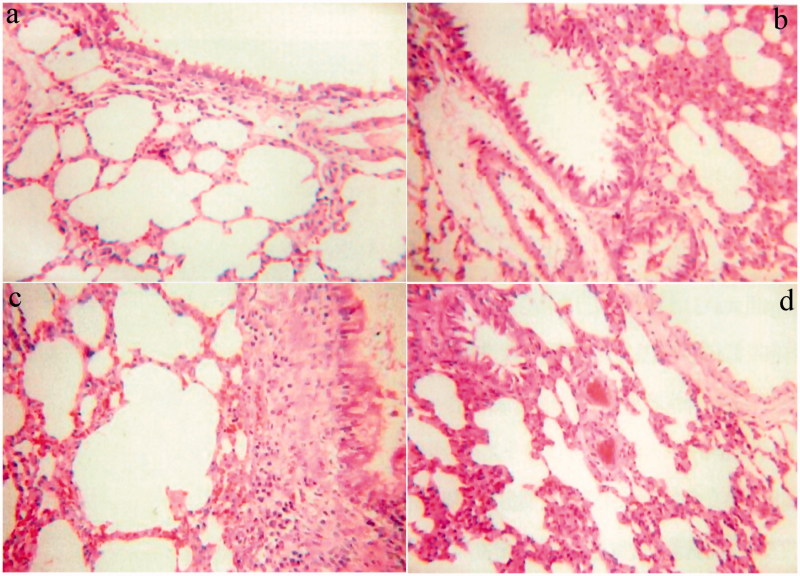
Representative photographs of lung tissue 24 h post-administration of the blank DPIs inhalation (a); the INS@FDKP-MPs inhalation (b); the Common INS inhalation (c); and the subcutaneous insulin injection (d) (magnification: ×200).

The safety of INS@FDKP-MPs was investigated by examining the irritation characterizations on the pathological section of the lung through a light microscope, such as congestion, necrosis, and inflammatory cell infiltration. As shown in Figure 4, in addition to the subcutaneous injection group, rats in all research groups showed mild lung irritation 24** **h after administration, but the INS@FDKP-MPs are less irritating to the lung than the common INS group which uses lactose as a carrier. The alveoli showed uniform size and intact structure with the thicknesses of alveolar cavity walls also kept normal in the INS@FDKP-MPs group. This indicated that the rapid absorption of INS@FDKP-MPs in the lungs can weaken the stimulation of the lungs, suggesting that the safety of pulmonary delivery of INS@FDKP-MPs was satisfied.

## Discussion

With the poor compliance and delayed effect time of subcutaneous injection, different insulin delivery routes were investigated to achieve the fast absorb behavior which could better simulate the insulin secret situations *in vivo*. Therefore, INS@FDKP-MPs dry powder inhalation was designed, prepared, and investigated to confirm the hypoglycemic effect.

The insulin was loaded on FDKP microparticles through electrostatic adsorption at pH 4.5. As described before, FDKP could rapidly dissolve in neutral or alkaline solutions while precipitated to form FDKP microparticles when pH was lower than 5.0 as the degrees of carboxyl dissociation decreased, which further decreased the solubility of FDKP. The microparticles surface shows strong negative charges which could use to load the proteins drug with positive charges on the surface. The preparation process of INS@FDKP-MPs was optimized and the insulin entrapment rate was higher than 95% with drug loading about 13%, which indicate that FDKP has a large enough loading capacity as a drug carrier for inhalation.

The INS@FDKP-MPs dry powders were prepared by spray drying with a yield higher than 50% and *D*_aer_ about 1.6** **μm. The particles size was key factors to affect the deposition. Specifically, inhaled particles with a *D*_aer_ large than 5** **μm will remain in the upper respiratory tract such as nose, mouth, or throat, moreover *D*_aer_ small than 1** **μm will be exhaled after initial inhalation which also shows ineffective deposition. Using laser particle analysis, the *D*_aer_ of INS@FDKP-MPs was found to be 1.6** **μm which can be considered as a good candidate for insulin deposited into the deep lung (Flament et al., 2004).

Flowability was also an indispensable part of the aerodynamic behaviors and was the macroscopical expression of the intergranular force of inhaled particles, which was characterized by the repose angle and Carr’s index. FDKP microparticles were self-assembled by microcrystallite and leading to a large number of empty space existed in the core or the surface of particles which further reduced the particle density (Vartiainen et al., 2016). The low moisture content in INS@FDKP-MPs also helps the reduction of inter-force between particles as a suitable amount of water could cover on the surface of particles and forming a water film which could reduce electrostatic friction between particles, while excess moisture could avoid the flowability as the friction between water layers (Courrier et al., 2002) .

SEM was used to visualize the surface morphology of FDKP microparticles and INS@FDKP-MPs. FDKP microparticles show rosette shape with porous on the surface which increase the specific surface area compare to solid microparticles and shows higher drug loading capacity (Cassidy et al., 2011). Insulin was filled in the pores or the cracks of the blank FDKP microparticles, leading to the smoother surface after the co-spray drying. XRD and DSC were used to confirm the interaction between insulin and FDKP.

To confirm the interaction between insulin and FDKP, the crystallinities of INS@FDKP-MPs were determined by XRD and DSC. Respectively, raw insulin exhibited an amorphous state and had no obvious diffraction peak, which was exemplified by a series of sharp 2*θ* peaks in the XRD pattern. For FDKP, a sharp peak was observed at 2*θ*** **=** **7°, 18–19°, and 25°, suggesting that FDKP can form a crystalline structure during precipitation at acidic conditions. The INS@FDKP-MPs shows same diffraction peak with FDKP microparticles spray dry powders with characteristic diffraction peak at 2*θ*** **=** **25° and small peaks at 2*θ*** **=** **7° and 18–19°, which indicated spray drying with insulin will not affect the crystal type of FDKP. And it also showed that insulin could produce certain inhibitory effect on FDKP in the spray drying process.

DSC was also used to further characterize the crystallinities of INS@FDKP-MPs. It showed that the endothermic peak of insulin and FDKP was respectively at around 70 and 340** **°C (represented the melting of FDKP). In the physical mixture of insulin and FDKP, there was no significant shift of the endothermic peaks of insulin and FDKP at 66.5 and 349.2** **°C. However, for INS@FDKP-MPs, during spray drying, the melting peak of it appeared at 343.1** **°C with the complete disappearance of insulin melting peak at 67.7** **°C, but a new melting peak appeared around 282.5** **°C, confirming the adsorption of insulin on the microsphere surface.

The aerodynamic properties of INS@FDKP-MPs were extensively investigated using NGI. The mechanisms by which aerosolized aerosol particles deposit in the lungs include diffusion, interception, gravity settling, and electrostatic effects (Cuvelier et al., 2015). During this process, drug deposition was affected by several factors such as particle properties, respiratory geometry, and breathing patterns. Among them, the particle size and its distribution mainly determine the amount of deposition in the lungs, which affects the efficacy of the drug. Therefore, it is important to analyze the particle size of the microparticles by NGI (Liang et al., 2006). In the results, the dry powder inhalation shows high deposition in stages 3 and 4, indicating that the powder could be deposited into deep lung with satisfied dispersion performance.

To evaluate the efficacy of pulmonary delivery of insulin against diabetes, INS@FDKP-MPs were administered in a rat model by intratracheal insufflation. A type I diabetes of rat model was established by streptozotocin (STZ), which was a glucosamine-nitrosourea and also a DNA alkylating agent that can enter cells individually through the GLUT2 glucose transport protein (Wenzel et al., 2008). It had a selective destruction effect on islet β cells of certain species of experimental animals and can induce diabetes in various animals such as rats and mice. Because STZ had less toxicity to the body tissue and led to high animals survival rate, it was a widely used drug for preparing animal models of diabetes at home and abroad (Savill et al., 1989).

The target of insulin was located at the muscle and liver, but the receptors on the cell membrane can only specifically recognize insulin monomers. After administration INS@FDKP-MPs by inhalation, the blood glucose concentration decreased after 5** **min, the onset time was comparable to that of subcutaneous injection. It was speculated that most of the insulin in the powder was in the form of a monomer so that the onset speed of the inhalation powder can be greatly accelerated. The results also showed that with the increase of the dose, the hypoglycemic effect was enhanced, and the peak time of different doses was prolonged with the increase of the dose. Compared with insulin for injection, the duration of the powder was shorter than that of subcutaneous injection and it had the characteristics of rapid elimination, which was closer to the secretion of human endogenous insulin and could reduce the occurrence of side effects such as hypoglycemia and weight gain.

In the deep lungs, the removal of foreign bodies is mainly carried out by the phagocytosis of macrophages (Wang et al., 2018). Phagocytosis causes macrophage to continue to activate, secreting large amounts of inflammatory mediators and further chemotaxis, activating polymorphonuclear cells (PMN), causing it to accumulate in the alveolar cavity-pulmonary capillaries, and eventually led to alveolar damage and increased capillary permeability. In order to study the pulmonary irritation of INS@FDKP-MPs on normal rats, the powder was investigated by exam the irritation characterizations on the pathological section of the lung through a light microscope. It showed that there was no evidently systemic or local irritation after pulmonary administration in rats. Excluding the influence of the operation, there was no significant difference between the powdered aerosol formulation and the lactose carrier in the administration group. It was speculated that the INS@FDKP-MPs enter the alveoli and absorb into the blood quickly, which reduce the residence of the powder on the alveolar surface, so that lessen the effects of macrophages weakening. It was also indicated that the rapid absorption of INS@FDKP-MPs in the lungs can weaken the irritating effect of inhaled powder on the lungs, and was suitable for pulmonary administration

## Conclusion

In this study, we have demonstrated the use of FDKP, approved by the FDA for insulin delivery. FDKP as a carrier material prepared insulin lung powder inhalation not only possess good reproducibility but also has good atomization performance and effective pulmonary deposition, which is an effective therapy for the diabetes treatment.

Results *in vivo* clearly suggested that insulin dry powder inhalations for targeted pulmonary delivery offer advantages in providing direct access to disease sites and minimizing the occurrence of side effects compared to subcutaneous injection insulin. INS@FDKP-MPs powder inhalation could rapidly decrease the blood sugar level without immune stimulation, which shows remarkably potential for diabetes treatment by pulmonary delivery route.

Irritation test results showed that 24** **h after administration, some symptoms like the trachea, bronchus, lung congestion were not observed. The alveoli show uniform size and intact structure with the thicknesses of alveolar cavity walls also keeps normal in all research groups, suggesting the safety of pulmonary delivery of INS@FDKP-MPs was satisfied.

In summary, this research demonstrated an innovative approach of formulating existing therapeutics for targeted pulmonary delivery to better manage diabetes. It also shed light on exciting opportunities to utilize existing technologies to overcome the limitations of conventional delivery routes, potentially improving both bioavailability and therapeutic efficacy.

## Supplementary Material

Supplemental Material
