# Supplementary material for: Preparation, characterization, and pharmacodynamics of insulin-loaded fumaryl diketopiperazine microparticle dry powder inhalation
Source: Drug Deliv. 2019 Jul 1;26(1):650–60. doi: 10.1080/10717544.2019.1631408 (PMC6609328; doi:10.1080/10717544.2019.1631408)
Supplement: Supplemental Material [file IDRD_A_1631408_SM5554.docx]

# SUPPLEMENTARY MATERIALS

Research on Preparation, characterization and pharmacodynamics of insulin loaded fumaryl diketopiperazine microparticle dry powder inhalation

Yu Xia et al

**Preparation, characterization and pharmacodynamics of insulin loaded fumaryl diketopiperazine microparticle dry powder inhalation**

Yun Xia^1,2#^, Yipeng Su^1,2#^,Qiyue Wang^1,2^, Chen Yang^1^, Baoqiang Tang^1,2^, Yue Zhang^1,2^, Jiasheng Tu^1,2*^, Yan Shen^1,2*^

^1^ Center for Research Development and Evaluation of Pharmaceutical Excipients and Generic Drugs, China Pharmaceutical University, 24 Tong Jia Xiang, Nanjing 210009, China

^2^ State Key Laboratory of Nature Medicines, Department of Pharmaceutics, China Pharmaceutical University, 24 Tong Jia Xiang, Nanjing 210009, China

^#^ These authors contributed equally to this work.

Correspondence: Yan Shen, Jiasheng Tu

Center for Research Development and Evaluation of Pharmaceutical Excipients and Generic Drugs, China Pharmaceutical University, 24 Tong Jia Xiang, Nanjing 210009, China

Tel +86 025-83271305

Email shenyan@cpu.edu.cn (Y. Shen); E-mail: [64918159@qq.com](mailto:64918159@qq.com) (J. Tu);

# Material and methods

***Synthesis of fumaryl diketopiperazine (FDKP)***

FDKP was synthesized using a three-step reaction (Figure S1). In the step 1, the trifluoroacetyl-diketopiperazine (TFA-DKP) was synthesized by amino acid cyclization reaction. Briefly, the composition of N6-trifluoroacetyl-L-lysine (TFA-Lys) and phosphorus pentoxide (P_2_O_5_) (TFA-Lys : P_2_O_5_= 5.682 : 1 by weight ratio) were dissolved in 15 mL of N-methyl-2-pyrrolidinone (NMP) and reacted at 160-170 °C for 1.5 h under continuous purging of nitrogen. The obtained reactant was poured into a large amount of deionized water, stirred, and then refrigerated in a refrigerator at 4 °C until the solid was completely precipitated, followed by suction filtration, the filter residue was repeatedly washed twice with deionized water, and the product was vacuum dried for 24 h to obtain TFA-DKP. FDKP-ethyl ester was synthesized by amidation reaction in the step 2. TFA-DKP was hydrolyzed under the conditions of 10 mL of 5 % K_2_CO_3_ alkaline aqueous solution in order to remove the trifluoroacetyl group, EDC and monoethyl fumarate were dissolved in 20 mL of DMF at a mass ratio of 2:1, and stirred to completely dissolve. Then, NHS was added and activated at 0 ° C for 2 h under nitrogen protection. The aqueous solution of DKP was then adjusted to pH 7-8 with acetic acid, added dropwise to the activated EDC solution and reacted overnight at room temperature. The mixture was concentrated by vacuum concentrator, and deionized water was added for precipitation, followed by suction filtration and dried for 24 h to collect the FDKP-ethyl ester. In step 3, the synthesized FDKP-ester was dissolved in 1 % NaOH water-methanol (1:1) solution for about 6 h at 25 °C, then the pH of the solution was adjusted to neutral with acetic acid and the methanol was removed by rotary evaporation at 40 °C. Finally, the pH was adjusted to acidity to allow for the precipitation of FDKP, the precipitates were collected by filtration. The structure of the product was confirmed by 1H-NMR (AVANCE AV-300, BRUKER, Switzerland) (solvent: DMSO-d6, frequency: 300 MHZ, temperature: 25 °C). Then, the FDKP was compressed with 1 % potassium bromide to obtain IR spectrum (TENSOR 27, Bruker, Germany). The FDKP was also dissolved in methanol and injected into the ion source through a sampling pump at a flow rate of 180 μL·h^-1^. The atomizing gas and drying gas was nitrogen, pressure was 10 psi, flow rate was 5 L·min^-1^, temperature was 250 °C, spray voltage was -4000 V and Helium gas was used as a collision gas in the secondary mass spectrometry to obtain an ESI-MS diagram ( Bruker Esqure 3000plu, Bruker, Germany).


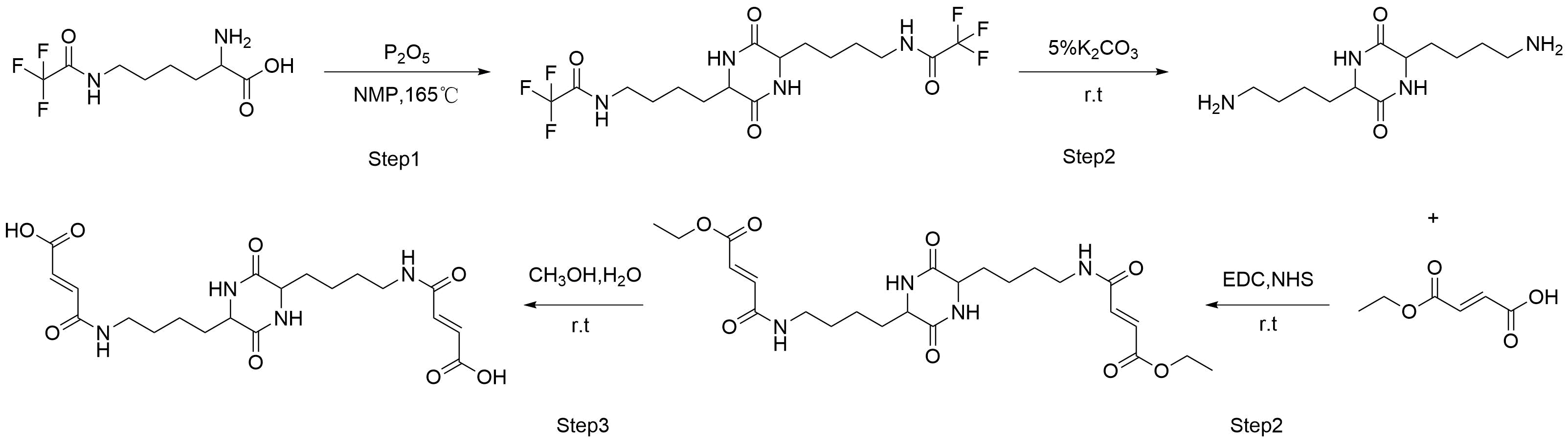


**Figure.S1 Synthesis of FDKP.**

***Orthogonal design optimization of spray drying process for the preparation of INS@FDKP-MPs***

Nine batches of INS@FDKP-MPs were prepared according to the orthogonal test table, the yield, aerodynamic diameter, moisture and angle of repose were taken as the main indicators and Z-comprehensive scoring method was used to evaluate and select the optimal spray drying preparation process, L_9_ (3^4^) orthogonal table was shown in Table S1.

Z-comprehensive scoring method was simple, intuitive and easy to calculate. The comprehensive index Z of this method is calculated by multiple indicators, which can make the calculation index non-dimensional. When calculating by this method, two indicators (e.g. high-quality and low-quality) were usually studied. For the “high-quality” indicator (such as yield), the larger the evaluation index value, the better; “Low-quality” indicators (such as moisture content, aerodynamic diameter, and angle of repose), the smaller the evaluation index value, the better.

**Table. S1 Factors and levels of spray-drying preparation parameters.**

| Levels | A | B | C | D |
| --- | --- | --- | --- | --- |
|  | Pump speed（%） | Air input（L·h^-1^） | Aspirator（%） | Inlet temperature（℃） |
| 1 | 4 | 466 | 80 | 120 |
| 2 | 6 | 533 | 90 | 130 |
| 3 | 8 | 601 | 100 | 140 |

# Results

***Synthesis and characterization of FDKP***

The signals at *δ*=8.04~8.11 ppm were attributed to the amide bond (-NHCO) proton peak, and *δ*=8.42~8.58 ppm corresponded to the amide bond (-NHCO) proton peak on the mother nucleus, indicating the formation of two amide bonds between the FDKP ring structure and the fumaric acid. A broad peak characteristic of carboxyl hydrogen (-COOH) occurred between *δ*=9.5 and 12.0 ppm, confirming that the ethyl ester in the FDKP-ester structure was hydrolyzed to obtain a carboxyl group to form FDKP (Figure S2A). The FDKP was further confirmed by the appearance of the stretching vibration peak of C=O at 1712 (*s*), the stretching vibration peak of CH=CH at 1673(*s*), and the characteristic absorption peak of amide bond NH-CO at 1539(*s*) in the IR spectrum (Figure 3B). Lastly, the mass spectrum (Figure S2B) demonstrated three peaks at m/z: 451 (100) (M-1), 473 (18) (M-2+Na) and 474 (5) (M-1+Na), which corresponded to the molecular peaks of FDKP (M_w_ 452). To sum up, these tests verified the successful synthesis of FDKP (Figure S2C).


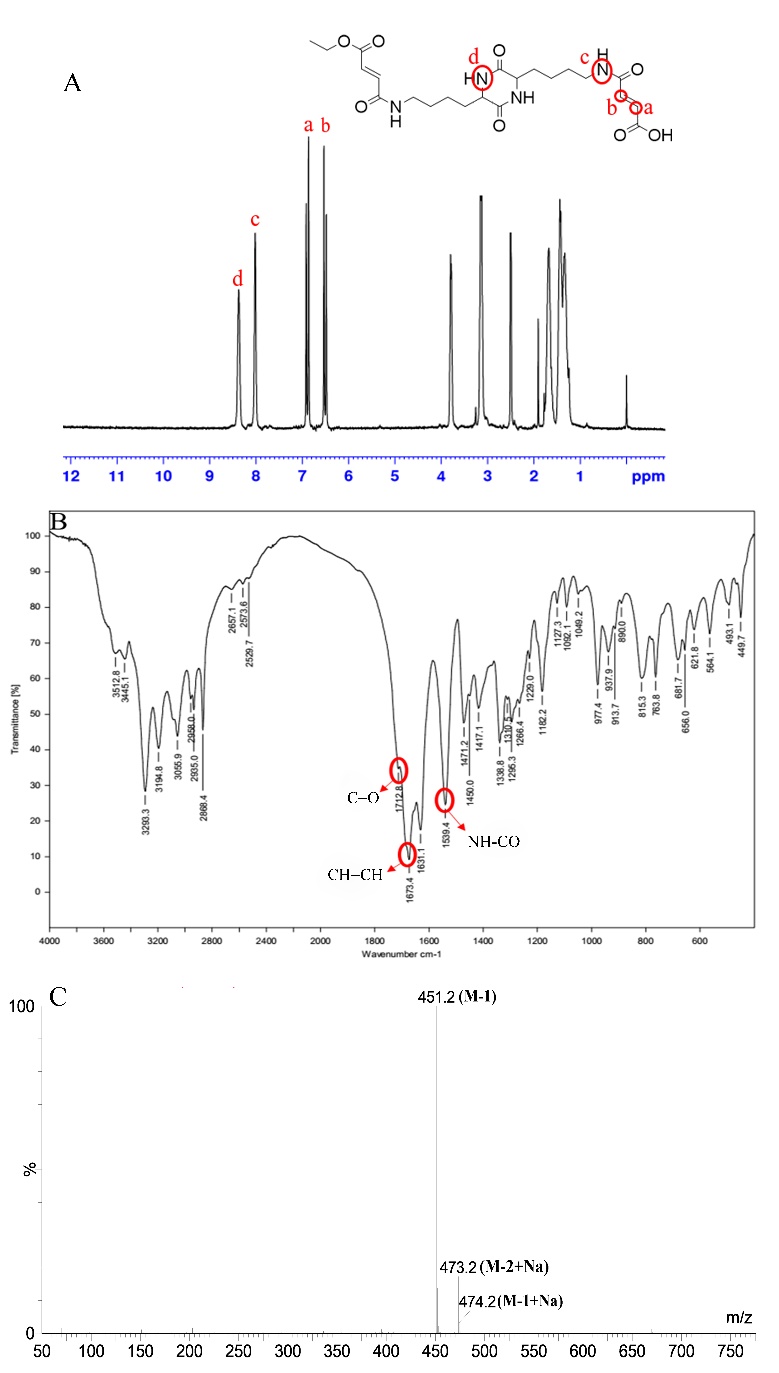


**Figure.S2 (A) The ^1^H-NMR of FDKP; (B) IR spectrum of FDKP; (C)The ESI-MS of FDKP**

***Results of spray-drying orthogonal experiment for preparation of INS@FDKP-MPs***

The calculation method of the Z-comprehensive scoring method is shown in formulas (1) and (2).

$Z_{i}=(X_{i}-\bar{X_{i}})/S_{i}$ (1) $\sum Z_{i}=\sum Z_{ih}+\sum Z_{il}$ (2)

In formula (1), it is the comprehensive score of a certain index, Xi is the index value, which is the average value, and Si is its standard deviation. When applying the formula (2) for calculation, the Z value of Z_ih_ is calculated as “addition”, and the Z value of Z_il_ is calculated as “subtraction”, which can be obtained. The larger the value, the better the condition, and the corresponding condition is the optimal spray drying process condition. The Z value is used as the index value to be substituted into the formula (2) for calculation. The comprehensive calculation result is shown in Table S2 and Table S3.

**Table.S2 Results of spray-drying orthogonal experiment.**

| No. | Field(%) | D_aer_(μm) | Moisture(%) | repose(º) |
| --- | --- | --- | --- | --- |
| 1 | 38.4 | 2.64 | 2.34 | 33.2 |
| 2 | 47.6 | 3.21 | 1.96 | 36.1 |
| 3 | 51.7 | 2.18 | 2.25 | 44.8 |
| 4 | 37.3 | 5.36 | 4.17 | 30.7 |
| 5 | 40.1 | 3.52 | 2.49 | 36.1 |
| 6 | 50.9 | 4.92 | 0.76 | 34.7 |
| 7 | 48.5 | 5.05 | 1.27 | 31.1 |
| 8 | 47.1 | 3.19 | 3.84 | 38.4 |
| 9 | 53.2 | 2.71 | 1.95 | 30.6 |

**Table.S3 Results of Z-score comprehensive evaluation method.**

| No. | A | B | C | D | Z-score |
| --- | --- | --- | --- | --- | --- |
| 1 | 2 | 1 | 2 | 3 | -0.021 |
| 2 | 1 | 1 | 1 | 1 | 0.742 |
| 3 | 3 | 3 | 2 | 1 | -0.133 |
| 4 | 3 | 2 | 1 | 3 | -3.651 |
| 5 | 3 | 1 | 3 | 2 | -1.261 |
| 6 | 1 | 2 | 2 | 2 | 1.248 |
| 7 | 2 | 3 | 1 | 2 | 1.059 |
| 8 | 2 | 2 | 3 | 1 | -1.558 |
| 9 | 1 | 3 | 3 | 3 | 3.329 |
| K_1_ | 2.245 | -1.329 | 0.357 | 3.085 |  |
| K_2_ | -0.713 | 2.171 | -1.518 | -0.731 |  |
| K_3_ | -1.532 | -0.842 | 1.161 | -2.354 |  |
| k_1_ | 0.748 | -0.443 | 0.119 | 1.028 |  |
| k_2_ | -0.238 | 0.724 | -0.506 | -0.244 |  |
| k_3_ | -0.511 | -0.281 | 0.387 | -0.785 |  |
| R | 1.259 | 1.167 | 0.893 | 1.813 |  |
